# Remediation of chlorinated aliphatic hydrocarbons (CAHs) contaminated site coupling groundwater recirculation well (IEG-GCW®) with a peripheral injection of soluble nutrient supplement (IEG-C-MIX) via multilevel-injection wells (IEG-MIW)

**DOI:** 10.1016/j.heliyon.2022.e11402

**Published:** 2022-11-03

**Authors:** Paolo Ciampi, Carlo Esposito, Ernst Bartsch, Eduard J. Alesi, Gert Rehner, Marco Petrangeli Papini

**Affiliations:** a Department of Earth Sciences, Sapienza University of Rome, Piazzale Aldo Moro 5, 00185, Rome, Italy; b IEG Technologie GmbH, Hohlbachweg 2, D-73344 Gruibingen, Baden-Württemberg, Germany; c Department of Chemistry, Sapienza University of Rome, Piazzale Aldo Moro 5, 00185, Rome, Italy; d CERI Research Center, Sapienza University of Rome, Piazzale Aldo Moro 5, 00185, Rome, Italy

**Keywords:** Hydrogeochemical conceptual modeling, Groundwater circulation well, Biological reductive dechlorination, Remediation strategy, Chlorinated aliphatic hydrocarbons, Nutrient injection

## Abstract

An innovative Groundwater Circulation Well (GCW) process was configured, installed, and tested for optimizing the distribution of a soluble nutrient supplement in a heterogeneous aquifer for reductive dehalogenation. This generated an in-situ bioreactor for the enhanced treatment of chlorinated aliphatic hydrocarbons (CAHs). At a site in Barcelona, Spain, trichloroethylene (TCE) concentration was found in the source area to a maximum value of up to 170 mg/L, while the degradation products like 1,2-dichloroethylene (1,2-DCE) and vinyl chloride (VC) were detected in significantly lower concentrations or were even absent. The novel system combined a vertical recirculation well (IEG-GCW®) and four multilevel injection wells (IEG-MIWs) to introduce the carbon solution into the aquifer. A 12 m deep IEG-GCW® equipped with 2 screened sections were located in the center of the 4 IEG-MIWs. The GCW induced flow moves the groundwater in an ellipsoidal recirculation cell to spread the supplements from the central GCW and from the peripheral MIWs in the aquifer body. Two multilevel sampling wells (IEG-MLSWs®) in the radius of influence (ROI) monitor the remediation process to capture hydrochemical variations along the vertical aquifer sections. A multi-source model harmonizes geological and hydrochemical information during different remediation stages, guiding the adaptation of the remediation strategy to physicochemical conditions and unmasking the decontamination mechanics induced by the remedial actions. Hydrochemical monitoring of MLWS and the stable carbon isotopic signature of cis-1,2-DCE and VC show the mobilization of secondary contamination sources triggered by recirculation during remediation, the stimulation of microbiological activity following nutrient supplement via GCW and MIWs, and the strong decrease of CAHs concentrations at different aquifer levels. Evidence from the first application at the field scale reveals a significant increase in the chloroethane biodegradation rate and short-term effectiveness of the innovative remediation strategy. GCW-MIWs synergy represents a promising strategy to degrade CAHs in a shorter period through the combination of a controllable hydraulic system, effective nutrient distribution, and the monitoring of the remediation process.

## Highlights

• Data-driven model tailors the remediation strategy to physicochemical constraints;
• GCW recirculation mobilizes CAHs and enhances 3D spread of additives;
• C-MIX redistribution via GCW-MIWs increases the rate and extent of bioremediation;
• Hydrochemical monitoring and CSIA reveal in situ biodegradative transformations;
• The novel strategy generates a long-term persistent hydro-geo-bio-chemical reactor.

## Graphical abstract

A pictorial summary of the main findings of the paper illustrating (1) the 3D geological model of the treatment area, (2) the remediation configuration impacting the chlorinated aliphatic hydrocarbon (CAH) plume in the stratigraphic framework, (3) the schematic layout of groundwater circulation wells (IEG-GCW®), multilevel injection wells (MIWs), and multilevel sampling wells (MLSWs), together with (4) hydrochemical evidence revealing both recirculation-induced mobilization of secondary contamination sources and stimulation of biological activity following biostimulant injection via MIWs.

**Figure:**
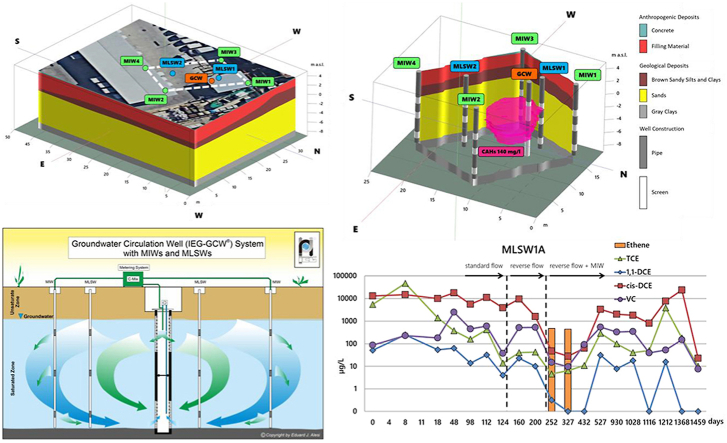

## 1 Introduction

Chlorinated aliphatic hydrocarbons (CAHs) represent the most frequently detected contaminants impacting groundwater ecosystems due to improper disposal or accidental spills (Nijenhuis et al., 2013; Blazquez-Pallí et al., 2019; Wang et al., 2020). Among these contaminants belonging to the class of dense non-aqueous phase liquids (DNAPLs), perchloroethylene (PCE), and trichloroethylene (TCE) are the most widely used substances in industrial applications (Rajajayavel and Ghoshal, 2015; Niño de Guzman et al., 2018; He et al., 2021). The removal of these toxic chemicals from the contaminated environment necessitates a deep understanding of the hydrogeological setting of the site, of the pollution scenarios, and dynamics as well as the transformations that these compounds experience once embedded in the hydrogeological and biochemical framework (Nijenhuis et al., 2013; Filippini et al. 2016; Blazquez-Pallí et al., 2019; Ciampi et al., 2019b, 2021a). Following a primary contamination episode, the redistribution of these pollutants in the environment, according to the scheme known as DNAPL architecture, arises from mainly vertical migration through the unsaturated zone along with retention, adsorption, and volatilization processes followed by dispersion and reactive transport in groundwater (Stroo and Ward, 2010; Kueper et al., 2014). The DNAPL distribution in the subsurface under different physical states (gaseous, dissolved, and separated phase) is influenced by the physicochemical characteristics of the porous medium. In the saturated domain, the mobility, transport, and distribution of DNAPL are governed by the hydraulic conductivity (k) of sediments (Brusseau and Guo, 2014). Low-permeability materials can first act as sinks to store pollutants when contaminant concentrations in adjacent high-permeability levels are high. Then they behave as secondary pollution sources via back-diffusion after contaminants are removed from coarse transmissive levels (You et al., 2020). Such secondary sources slowly release contaminants following the reversal of the concentration gradient by molecular diffusion (Yang et al., 2018). Source aging and back-diffusion processes generate residual contamination and plumes which may persist for decades (Mateas et al., 2017; Yang et al., 2018; Tatti et al., 2019). Besides, Puiserver et al. (2022) showed that heterogeneity and textural contrasts between fine and coarse grain size media control the distribution of secondary sources of DNAPL contamination and favor microbial development. In addition to physicochemical phenomena, biodegradative mechanisms triggered by microbial communities play a crucial role in the transformation and reduction of CAHs (Wang et al., 2015; Dolinová et al., 2017). Sequential reductive dechlorination of CAHs, operated by specific bacterial genera under a wide range of environmental conditions, converts PCE to TCE, dichloroethylene (DCE) isomers, VC (vinyl chloride), and ethene (Aulenta et al., 2007; Němeček et al., 2017; Wang et al., 2020; Xiao et al., 2020). Reductive dechlorination of PCE is favored by anaerobic conditions, while reductive dechlorination of TCE, DCE, and VC generally occurs under more reduced conditions (Dolinová et al., 2017; Puigserver et al., 2022). *Desulfitobacterium*, *Clostridium*, *Dehalobacter*, *Dehalococcoides*, and *Geobacter* are well-known genera of microorganisms able to reductively dechlorinate chlorinated aliphatic compounds (Anam et al., 2019; Blázquez-Pallí et al., 2019; Chen et al., 2018; Merlino et al., 2015; Puigserver et al., 2016).

Specific hydrostratigraphic and physicochemical conditions influence mobility, pollutant transformations, and decontamination mechanisms, defining the conceptual site model (CSM) (Ciampi et al., 2022a). Packages of big-data can synthesize information that differs in nature or resolution and lead to the generation of a multi-source CSM in which a huge volume of multidisciplinary data acquired during the characterization and remediation phases converges (Wycisk et al., 2013; Ciampi et al., 2019a, 2019b; Utom et al., 2019). Knowledge of CSM is a prerequisite for planning an environmentally sustainable remediation action and interpreting the impact of remediation efforts (Ciampi et al., 2021a; 2021b).

Conventional remediation strategies for CAHs-contaminated groundwater comprise physicochemical technologies such as pump and treat, direct-push-injections, reactive permeable barriers, and in situ chemical oxidation/reduction (Stroo and Ward, 2010; Brusseau and Guo, 2014; Kueper et al., 2014; Rajajayavel and Ghoshal, 2015; Niño de Guzmán et al., 2018; Guan et al., 2020; Ciampi et al., 2021b). In the last years, bioremediation of CAHs has gained attention in both research and full-scale remediation for its cost-effectiveness and less invasive nature than more traditional physicochemical methods (Blazquez-Pallí et al., 2019; Rossi et al., 2021). Microbial degradation of CAHs in an aquifer could occur through anaerobic reductive dehalogenation (Aulenta et al., 2007; Xiao et al., 2020). However, those reactions are usually slow and require the addition of a carbon substrate behaving as an electron donor to enhance the biological process (Aulenta et al., 2007; Dolinová et al., 2017; Pierro et al., 2017; Amanat et al., 2021). Direct subsurface injection of various reactive electron donors near pollution source areas to biostimulate the dehalorespiring bacteria poses a promising alternative approach for the rapid in situ remediation of aquifers (Niño de Guzmán et al., 2018; Schaefer et al., 2018). Although, direct agent injection tends to suffer from restricted mixing in the heterogeneous, anisotropic, and low permeable hydrogeologic environment (Dyer et al., 2006; Manoli et al., 2012). The above obstacles could be overcome through the installation of a groundwater circulation well specially designed by IEG Technologie GmbH (IEG-GCW®). The IEG-GCW® is an innovative solution that generates an in situ vertical groundwater circulation cell downward or upward by extracting from and re-injecting groundwater into several screen sections of a multi-screened well (Herrling et al. 1991a, 1991b, 1993a, 1993b; Bott-Breuning and Alesi, 1993; Stamm, 1997). Previous studies suggest that vertical hydraulic gradients triggered by the recirculation system induce mobilization of DNAPLs from secondary contamination sources (Petrangeli et al. 2016; Pierro et al., 2017; Ciampi et al., 2019a, 2021a; Tatti et al., 2019). Also, forced convection can effectively distribute supplements, stimulating dechlorination activity within the radius of influence of the well (Xia et al., 2019). For effective microbial degradation, groundwater circulation flow may develop optimal conditions for improved contact, throughout the interstices of the porous medium, between microbes, contaminants, groundwater, and carbon substrate (Ciampi et al., 2019a; 2021a).

Characterizing and monitoring in situ biodegradation within the hydrogeochemical framework can be addressed by a variety of techniques (Schaefer et al., 2018; Blazquez-Pallí et al., 2019; Ciampi et al., 2021b; Flores Orozco et al., 2021). Monitoring of CAHs concentrations can provide detailed evidence of contaminant abatement. However, hydrochemical measurements alone do not enable the distinction of physical attenuation mechanisms such as dissolution, adsorption, or dispersion from biological processes (Nijenhuis et al., 2013; Blazquez-Pallí et al., 2019). In this sense, compound-specific isotope analysis (CSIA) has been suggested as a more reliable and increasingly used method to assess biodegradation of chlorinated ethenes and monitor the in-situ transformation of chlorinated solvents by identifying characteristic isotopic signatures during microbial reductive dichlorination (Bloom et al., 2000; Slater et al., 2001; Hunkeler et al., 2005; Puigserver et al., 2020; Herrero et al., 2021).

Here we present a novel approach which involves some solutions developed by IEG Technologie GmbH to improve the distribution of an organic carbon solution (IEG-C-MIX) combining peripheral multilevel injection wells (IEG-MIWs) and an IEG-GCW® (Herrling et al., 1991a, 1991b; Stamm, 1997; Ye et al., 2021). An IEG-GCW® system consists of a well equipped with at least two or more hydraulically separated screen sections. Groundwater is extracted from one section and re-infiltrated into another section above or below (Herrling et al., 1991a, 1991b; Stamm, 1997; Ye et al., 2021). This creates a three-dimensional forced circulation around the well to increase the number of flushes through the pore space and enhance the release of pollutants from fine-grained sediment structures (Ciampi et al., 2019a, 2021a; Tatti et al., 2019). The water flowing into the well rotates several times within the radius of influence (ROI) before it leaves GCW (Stamm, 1997; Xia et al., 2019). Such forced recirculation flow has been exploited to distribute reagents in the subsurface, attract pollutants into the bioreactive zone near the well, improve the contact between pollutant and reagent, and enhance the dechlorinating microbiological action. At an industrial area of Barcelona (Catalonia, Spain), the chlorinated compound TCE and its metabolites 1,2-dichloroethylene (1,2-DCE), 1,1-dichloroethylene (1,1-DCE), and VC have been detected at concentrations up to 170 mg/L. For this reason, an IEG-GCW® system has been combined with a reagent/biostimulant metering system to mix and directly infiltrate an electron donor releasing amendment (C-MIX), for reductive degradation, into different screened intervals of GCW and peripheral IEG-MIWs. Multilevel sampling wells delivered by Technologie GmbH (IEG-MLSWs) are used to collect multiple, undisturbed groundwater samples along with the vertical profile for hydrochemical and CSIA (Bloom et al., 2000; Slater et al., 2001; Hunkeler et al., 2005; Petrangeli et al. 2016; Ciampi et al., 2019a, Ciampi et al., 2019b; Ye et al., 2021). The primary motivation of this paper was to investigate the application of a novel combined strategy to redistribute supplements in aquifers for rapid in situ bioremediation. Hence, the main objectives of this work are to (1) verify the increase in GCW-induced mobilization and solubilization of contaminants, (2) evaluate the effectiveness of the system in terms of biostimulant distribution, (3) assess the impacts of the coupled strategy for enhancing dechlorinating activity and reducing organochlorine compounds, (4) delineate the mechanisms and dynamics of decontamination in the hydrogeological and biochemical environment. The paper advanced knowledge on the interconnection between hydrostratigraphic and physicochemical conditions to govern and control the development of an effective remediation strategy.

## 2 Materials and methods

**Figure 1:**
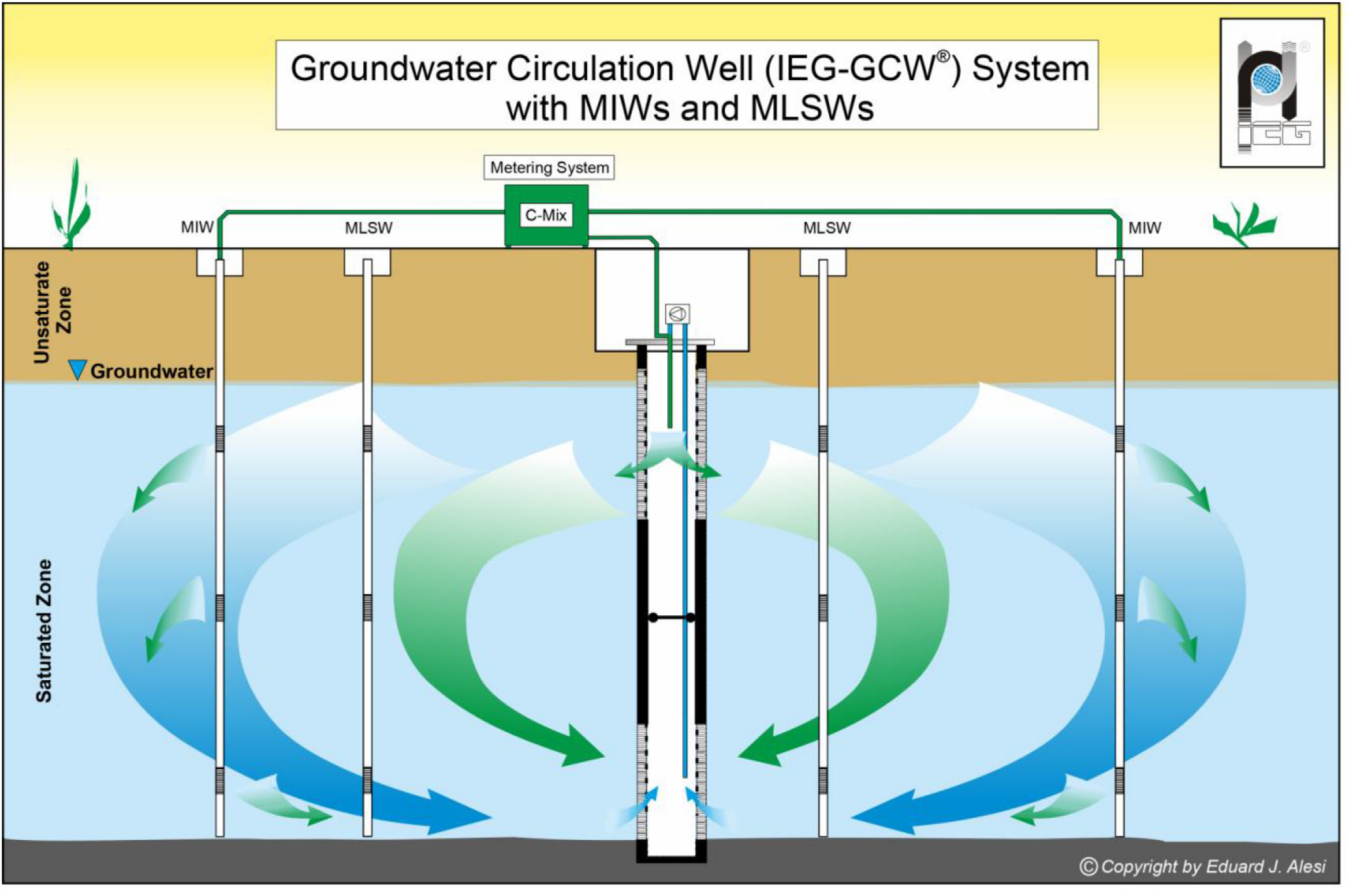
Schematic layout of the IEG-GCW® induced recirculation cell, IEG-C-MIX metering system, IEG-MIWs, and IEG-MLSW. Nutrient injection through GCW and sequential top-down injection via MIWs.

An IEG-GCW® combined with the addition of IEG-C-MIX was installed to enhance the biological dechlorination activity and reduce the concentrations of organochlorine compounds in the groundwater. The IEG-C-MIX, a nutritive preparation consisting of alcohols, saccharides, proteins, vitamins, and minerals had the scope to behave as a biostimulant. The mixture is composed of glycerol, molasses, and vitamin B12. Four multiple injection wells (IEG-MIWs) were drilled to introduce nutrients into the subsurface. An IEG-GCW®, 12 m deep and screened at 2 sections, was located in the center of the four IEG-MIWs. The nutrients were injected both centrally into the IEG-GCW and laterally via peripheral MIWs to address a more controllable and effective horizontal and vertical distribution and to speed up the remediation process. The extraction and re-injection of groundwater in the two different screen sections of the IEG-GCW® aimed to induce the generation of ellipsoidal groundwater recirculation cells with an orientation perpendicular to the well axis, potentially promoting the distribution of injected nutrients. The average recirculation flow rate was set to approximately 0.5 m^3^/h. Two submersible pumps with a power of 0.37 kW were used for the suction and distribution of groundwater in the two screened sections. Modification of the recirculation configuration enabled the generation of top-to-bottom (standard flow) and bottom-to-top (reverse flow) oriented recirculation cells, with the intent of investigating possible secondary source mobilization and contaminant abatement effects. Two IEG-MLSWs served for collecting undisturbed water samples, monitoring the remediation process, and capturing hydrochemical peculiarities at different depth intervals along the vertical (Figure 1).

The realization of the IEG-MIWs, IEG-MLSWs, and IEG-GCW® was preceded by the realization of stratigraphic logs and the installation of a piezometric monitoring network, to adapt the construction characteristics of the wells with a different design to the site-specific hydrogeological peculiarities. Twenty geological boreholes and thirteen piezometers support local hydrostratigraphic characterization. Most of the investigations' depths vary between 2 m and 13 m, except for one borehole that reaches 40 m. During the characterization phase, eleven core samples were collected for grain size analysis and estimation of the shallow aquifer permeability that is relevant for the contamination issue (see Figure S1, S2, and Table S1 of Supplementary Material). Piezometric level measurements were made on the network's thirteen piezometers, GCW, MLSWs, and MIWs. The installation of piezometers and wells over time resulted in a progressive increase in groundwater monitoring points. Some groundwater and soil chemical analyses were performed before the remediation (see Tables S2 and S3 of Supplementary Material). 12 soil samplings were collected for chemical analysis at different depths to determine CAHs concentration using headspace GC-MS. Groundwater was sampled at the piezometric network points with a submersible pump and analyzed in the laboratory via the GC-MS method to measure concentrations of chloroethenes. Groundwater characterization analyses were also collected from the treatment startup, covering a time interval of approximately four years. Also, groundwater and soil for a microcosm experiment were sampled from the most contaminated area (around PZ1). The soil used for the experiment was from the saturated zone (4.1–4.7 m). The microcosm was performed in a 120 mL serum bottle containing 15 g of soil and 50 mL of groundwater. Initially, 1,1-DCE, trans-DCE, cis-DCE, TCE, and PCE were added with a syringe through the cap at a nominal concentration of 2 mg/L of each compound. The microcosm test was conducted for 180 days to evaluate the intrinsic microbiological dehalorespiring activity in the soil and/or water of the site. Next-generation sequencing (NGS) experiments of bacterial 16S rDNA were performed at the end of the microcosm experiment to characterize the bacterial community of the site (Matturro et al., 2016). Lithostratigraphic and hydrochemical data gathered during the characterization phase were stored in a multiple excel worksheet, constituting a composite big-data package. Geological and hydrogeochemical parameters were interpolated with the inverse distance weighting algorithm, employing a number of neighboring points generally equal to four and a weighting exponent of two. Additional geoprocessing options included a high-fidelity filter to honor the control point value and light smoothing on surfaces (Ciampi et al., 2022a). Spatial interpolation of point data had for the purpose the generation of a geological and hydrochemical multi-source model, which served as a device to merge and interpret multi-modality information during the remediation phases. The hydrogeochemical model was developed in the domain of a three-dimensional voxel-based mesh, characterized by a node resolution of 1 m × 1 m × 0.2 m in the x, y, z directions respectively. The solid model consisted of a voxel number of 98 (x) × 90 (y) × 199 (z) and spanned from −34.8 to 4.8 m a.s.l. The elaboration of a 3D conceptual site model, employing a multiscale geomodeling approach, and the extraction of the geo-referenced information contained therein had the purpose of delineating secondary contamination sources and adapting the configuration of the remediation technology to the site-specific geological and physicochemical peculiarities. Hydrochemical monitoring of IEG-MLSWs and traditional piezometers, together with some isotopic analyses aspired to reveal decontamination dynamics, physicochemical, and biological processes triggered by recirculation system with different configurations and nutrient injection through IEG-MIWs and IEG-GCW. In the two monitoring campaigns following biostimulant injection, the analysis set included ethene measurements to gain insights into the biodegradative mechanisms triggered by microbial communities in the transformation and reduction of CAHs. CSIA was performed at different stages of the remediation to define the stable isotope ratios (δ^13^C) of chlorinated solvents and identify the main processes at play, such as degradation processes and potential evolution of the contamination, both spatially and temporally. The building of a big-data package and a multi-source, multi-temporal data-driven conceptual model has the goal of unmasking the evolutionary behavior of contaminants as a function of the adopted innovative strategy application, evaluating the impacts and efficacy of the combined physicochemical and microbial approach.

## 3 Results and discussion

### 3.1 The conceptual site model

**Figure 2:**
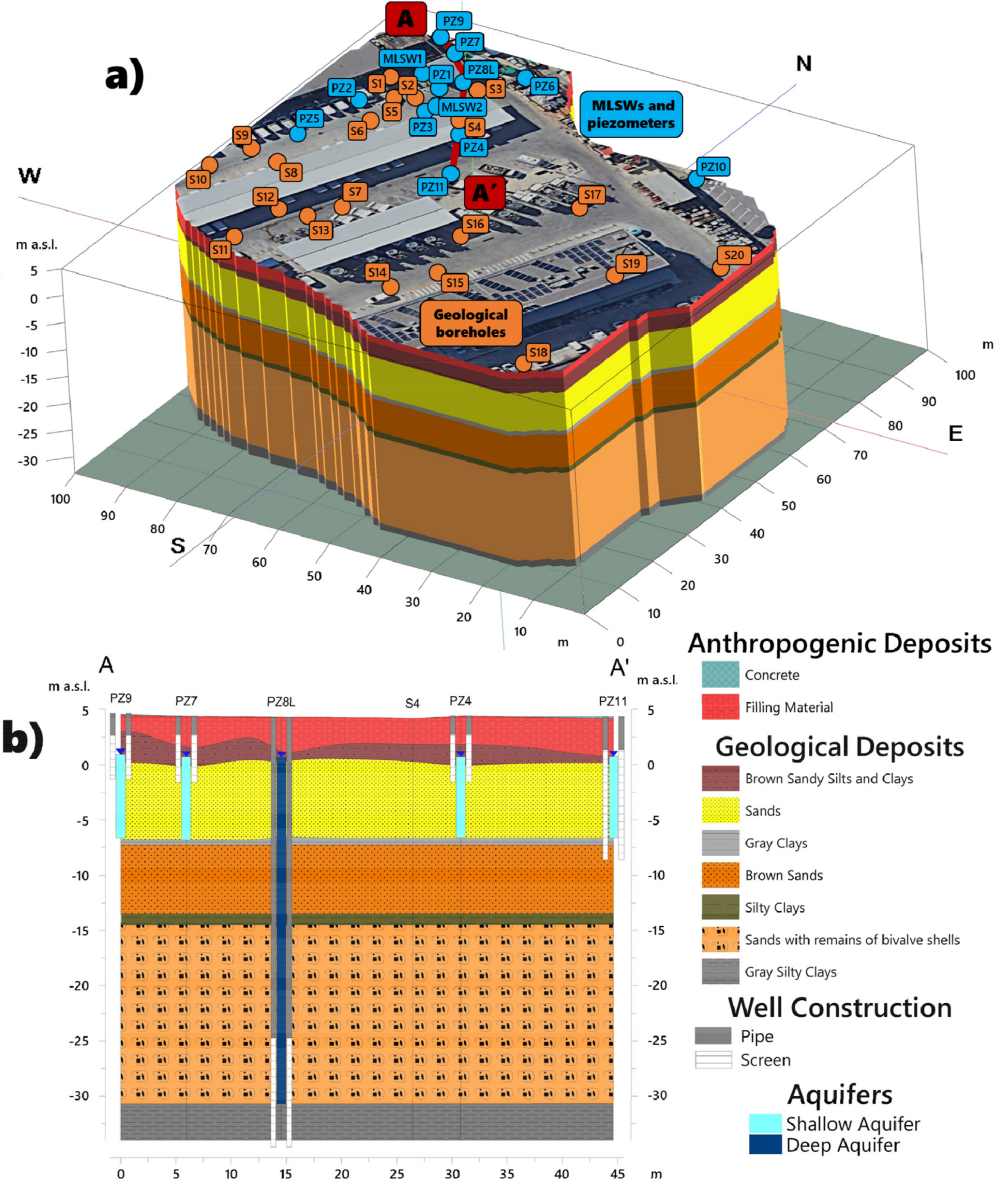
Three-dimensional stratigraphic model of the site showing the location of geological boreholes, monitoring wells, piezometers, and intersect line A-A' (a). Vertical profile A-A′, with superimposed information of geology, well depth, and groundwater level measured in different aquifer bodies (b).

The recent Quaternary unit that outcrops in situ consist primarily of sandy and silty sediments, gray to yellow. It comprises beach sediments, deposits of alluvial, prodeltaic, pre-delta, and deltaic origin. The upper portion of this unit presents anthropogenic fill (Filbà et al., 2016). The hydrostratigraphic model of the site depicts the stratigraphic relationships of the various lithotypes encountered in sequence up to a depth of 40 m (Figure 2a). Beyond the sporadic presence of concrete and filling material, the alternation of coarse-grained (sands) and fine-grained geological bodies (silts and clays) determines the fragmentation of the underground water circulation, delineating a multi-layer aquifer system. The limited thickness and uncertain spatial continuity of the low-permeability silty-clay layers separating the aquifer bodies suggest the potential communication of the subsurface aquifers (Renard and Allard, 2013). Gray clays, bounding the unconfined shallow aquifer, are found with continuity at a depth of about 11.5 m throughout the study area. A 1 m thick silty clay horizon was recognized by drilling borehole PZ8L at a depth of 18 m. Uncertainty about the spatial continuity of this layer suggests communication of groundwater circulation in the sandy sediments found at depths between 19 m and 35 m. The latter hosts the deep confined aquifer (Figure 2b).

**Figure 3:**
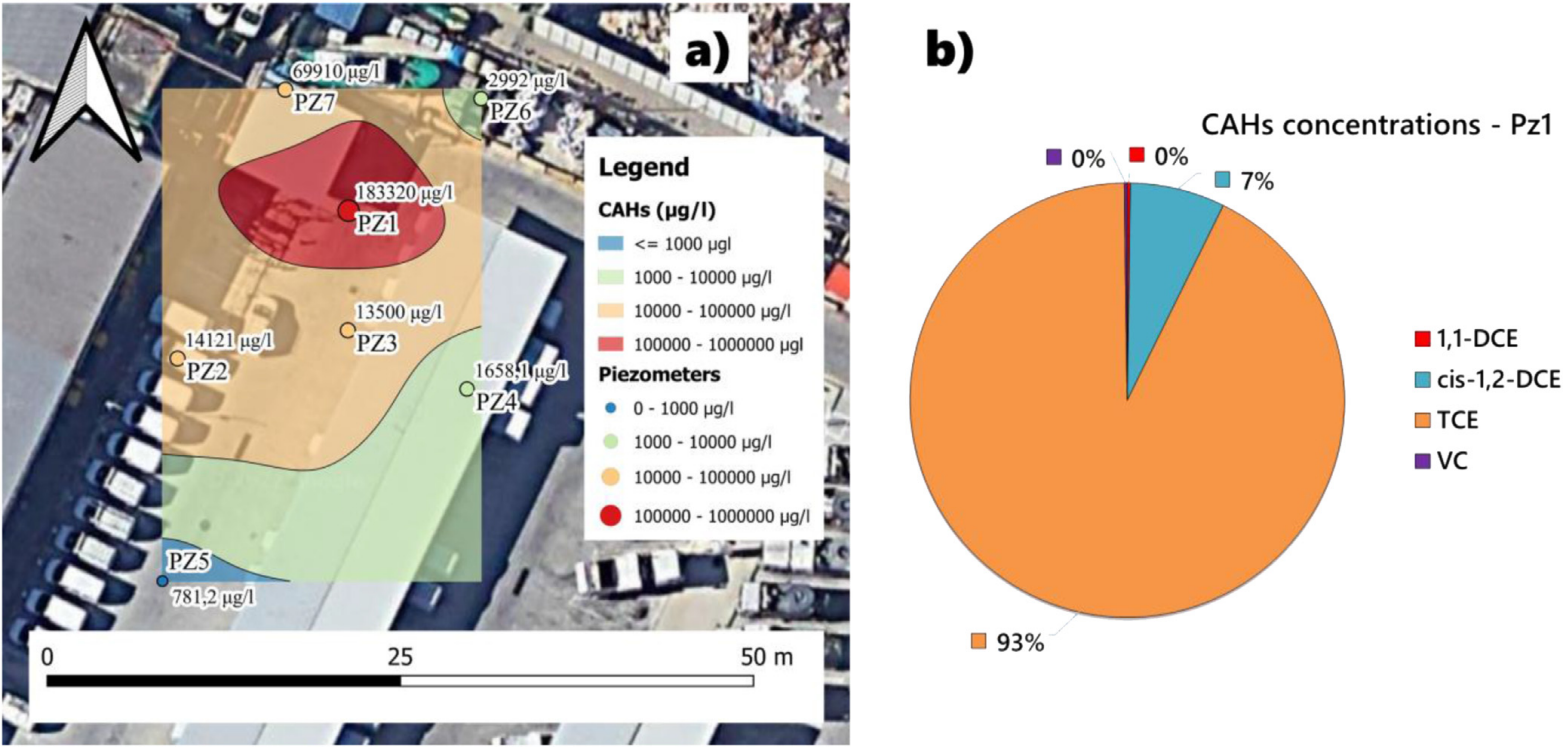
Contour map depicting the CAH contamination of the shallow aquifer in the pre-remediation monitoring campaign (a). Pie chart summarizing the ratio of CAHs detected in groundwater at point PZ1 (b).

Particle size analyses provide a detailed characterization of the shallow aquifer saturated horizon (see Figure S1, S2, and Table S1 of Supplementary Material). The shallow aquifer, approximately 7–8 m thick, consisting of heterogeneous alternating fine to coarse sands and locally silty-clayey layers with varying permeabilities between 1.44 × 10^−4^ m/s and 8 × 10^−5^ m/s, exhibits significant concentrations of CAHs in groundwater in a well-defined portion of the site (Figure 3a). Groundwater analyses for the pre-remediation monitoring campaign show highly chlorinated compounds (i.e., TCE, up to 170 mg/l), while 1,2-DCE, VC, and 1,1-DCE are detected only at low concentrations or are even absent (Figure 3b and Table S2 of Supplementary Material).

Although the concentration of 170 mg/L measured in PZ1 is about 13% of TCE solubility in water (1349 mg/L; Laor et al., 2003), DNAPL in a separate phase was not detected in piezometers of the monitoring network. The high concentrations in groundwater may be linked to variable hydraulic conditions induced by dynamic sampling. These cause the dissolution of residual and trapped DNAPL ganglia, leading to contaminant mobilization. Anderson et al., (1992) show that concentrations even close to saturation result from the rejuvenation of dissolution processes due to perturbations of groundwater flow. Sediment analyses (see Table S3 of the Supplementary Material) spatially delineate the secondary sources of contamination in the unsaturated domain, where chloroethenes are adsorbed onto solid subsurface materials. A fraction of the DNAPL that previously penetrated into the unsaturated zone would have reached the aquifer and remained as residual DNAPL (i.e., immobile and discontinuous DNAPL droplets or ganglia) trapped interstitially in silty-clayey deposits of the shallow aquifer, where significant contrasts in hydraulic conductivity occur (Puigserver et al., 2016, 2022). Heterogeneity in granular textures could favor the accumulation of immobile DNAPL phase formed by discontinuous nodules retained by capillary forces which are not affected by groundwater flushing and may be impacted by the alteration of groundwater hydrodynamics (Ciampi et al., 2021a; 2022b).

In this complex scenario, microcosm test and NGS experiments of bacterial 16S rDNA reveal the occurrence of several dechlorinating bacteria (see Figure S3 of Supplementary Material). The microbial community exhibits a high enrichment in *Geobacter sp.* (47% of total sequences). *Desulfovibrio sp*. accounts for 4.3% of total sequences. Both microorganisms have been described as PCE and TCE dechlorinating bacteria (Chen et al., 2018; Puigserver et al., 2016, 2022). Although *Dehalococcoides sp.* has been described as the only genus capable of completely degrading cis-DCE and VC in the nontoxic compound ethene, it accounts for only 0.9% of the total sequences in the microcosm (Anam et al., 2019; Blázquez-Pallí et al., 2019). *Clostridium* cluster *XIVa* represented over 10% (10.8%) of total sequences. Some Clostridiales have been reported to have some dehalorespiring activity as well, although no dechlorinating activity has been reported so far for *Clostridium* cluster *XIVa* (Merlino et al., 2015). The above evidence combined with the complete degradation of all compounds except trans-DCE at the end of the microcosm test (not shown here) delineates the intrinsic microbiological dehalorespiring activity in the soil and/or water of the site. These findings support the adoption of a remediation strategy for the biostimulation of autochthonous microorganisms.

### 3.2 The multisource model: an alignment and optimization tool for remediation strategy

**Figure 4:**
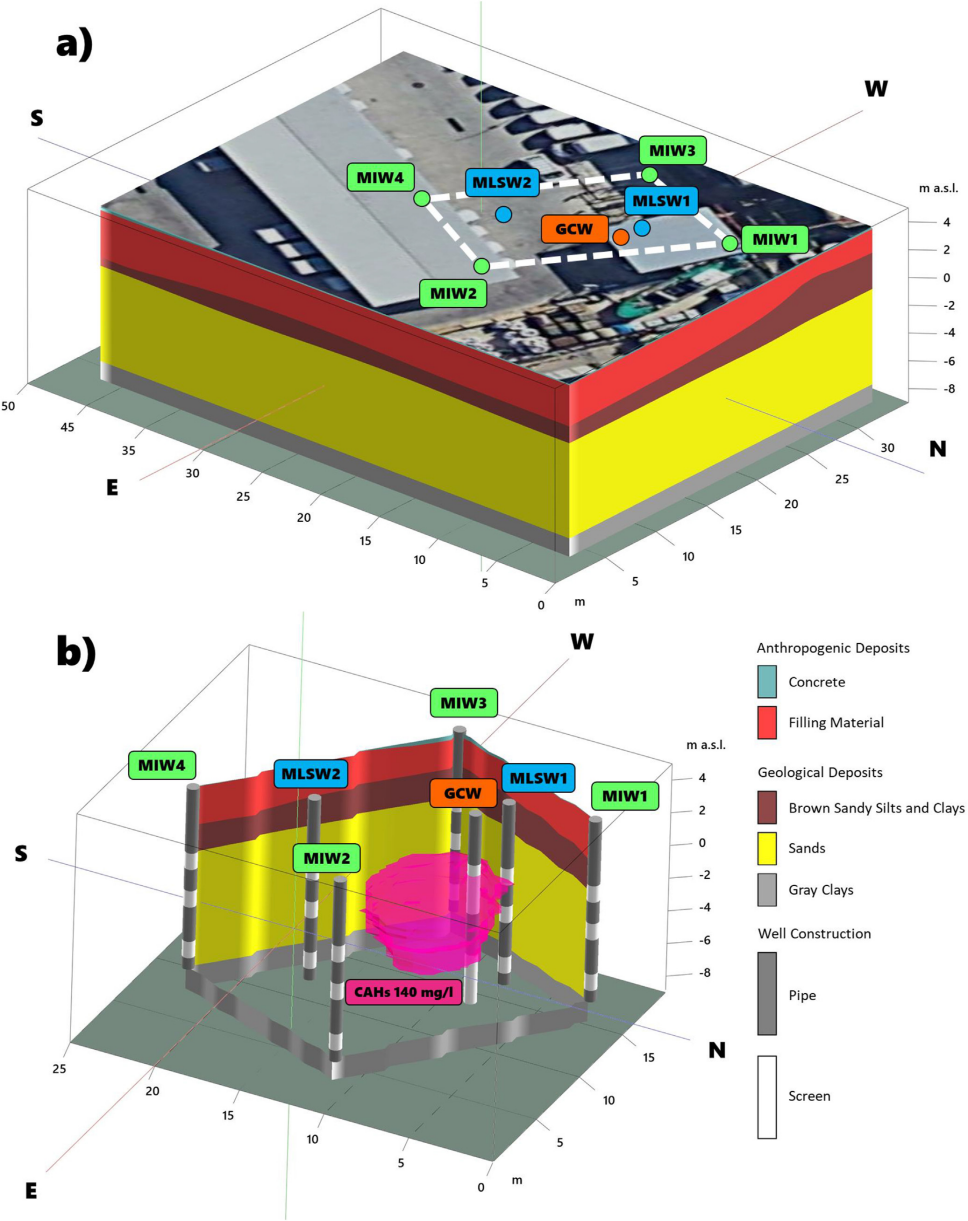
3D geological model of the treatment area and location of the remediation wells (a). Three-dimensional fence diagram illustrating the remedial configuration, the screening of the remediation and sampling wells in the stratigraphic framework, and the CAH concentration isosurface of 140 mg/l (b).

The multisource model synthesizes and harmonizes simultaneously information of different nature (geological, hydrochemical) and displays the hot spot with high CAHs concentrations in the NW portion of the site. The 3D conceptual model delineates the high CAH concentrations in the geological background of the shallow aquifer that is impacted by residual contamination. Data extraction in the spatial domain of the shallow aquifer supports the arrangements of the remediation wells. The positioning of GCW and MIWs reflects site-specific conditions to effectively target the contamination source while MLSWs control the effectiveness of the installed system (Figure 4a and b).

**Figure 5:**
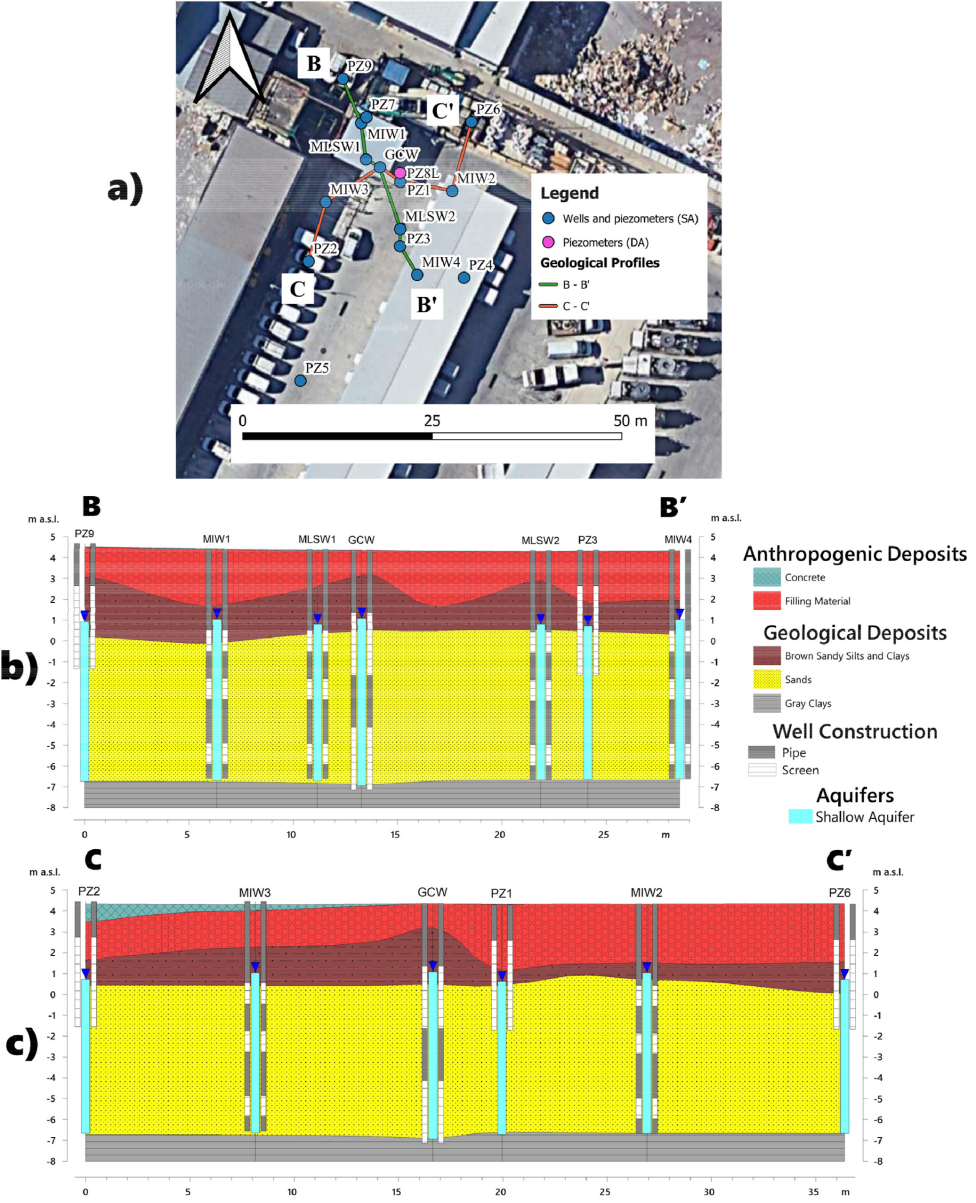
Map of geological profiles illustrating the location of piezometers intercepting the shallow aquifer (SA) and the deep aquifer (DA) (a). Geological cross-sections depicting the groundwater level, the screens of the remediation and sampling wells in the stratigraphic framework of the shallow aquifer (b–c).

The hydrogeological sections in Figure 5a, b, and c provide a two-dimensional view of the treatment configuration and screen locations in relation to geology and saturated aquifer thickness.

The groundwater table is situated 3.5 m below ground level, while the aquifer bottom lies at a depth of approximately 12 m. The arrangement of MIWs with three screened sections at depths of 4–5 m, 6–7 m, 9–10 m enhance the effective distribution of C-MIX in the aquifer body (Ye et al., 2021). The GCW screen sections at depths from 3-6 m and 8.5–11.5 m develop the vertical circulation cell for flushing the contamination source. A comparison of groundwater levels in the pre-remediation phase and following GCW activation in standard-flow mode (see Figure S4 of the Supplementary Material) reveals the perturbation of the equilibrium state of groundwater flow and the local increase of hydraulic gradients over an ROI of about 17 m.

### 3.3 The hydrochemical evidence disclosing decontamination mechanisms

**Figure 6:**
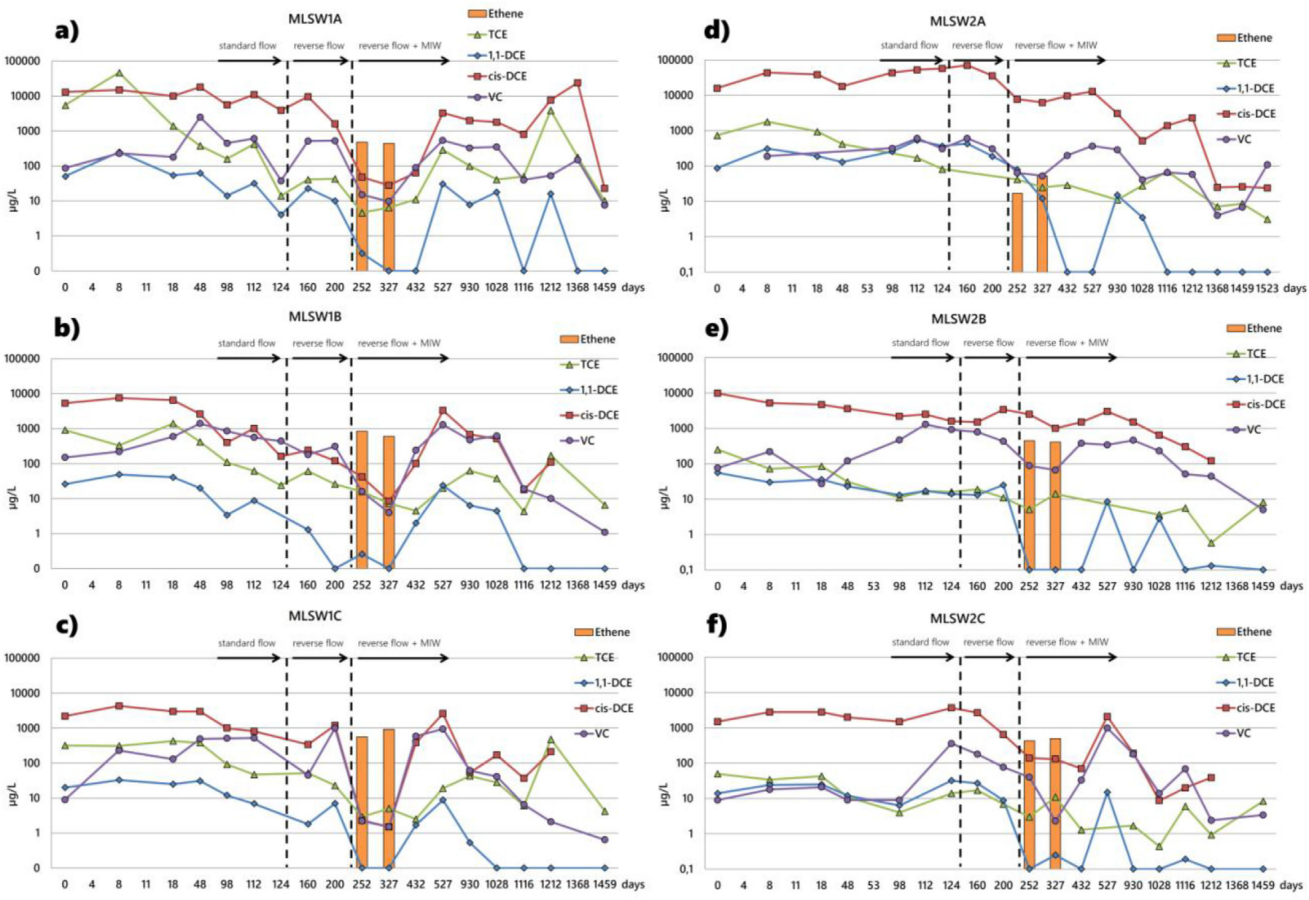
Hydrochemical monitoring of CAHs concentrations (log scale) measured at different depth intervals A (4–5 m), B (6–7 m), C (9–10 m) during remediation time for MLSW1 (a, b, c), and MLSW2 (d, e, f) sections.

MLSW1 and MLSW2 at a distance of 2.5 m and 8 m within the GCW influence radius delivers insights into the decontamination dynamics triggered by both recirculation and injection of reagents, at different sampling intervals along with the vertical profile: A (4–5 m), B (6–7 m), C (9–10 m). Hydrochemical monitoring of MLWSs reveals the mobilization of secondary contamination sources induced by recirculation with different configurations (standard and reverse flow) and the stimulation of biological activity following nutrient injection via MIWs. Indeed, the addition of C-MIX results in the formation of ethylene and unequivocally testifies to the stimulation of in situ dechlorinating activity (Figure 6a, b, c, d, e, and f).

Groundwater sampling and analysis indicate that dissolved oxygen (DO) and oxidation-reduction potential (ORP) decrease with reverse recirculation and biostimulant injection. The ORP measured in the three sampling ports of MLSW1 and MLSW2 exhibits an average value of −0.6 mV and 8.5 mV in the standard flow configuration, respectively. The reverse flow configuration results in a decrease in the average value of the redox potential along the sampling verticals of MLSW1 (−167.8 mV) and MLSW2 (−118.1 mV). Injection of C-MIX in a reverse flow pattern reveals an average ORP of −133.9 mV and −109.8 mV in MLSW1 and MLSW2, respectively. Detailed information on groundwater hydrochemistry at different stages of remediation is given in Table S4–S9 of the Supplementary Material. These refer to the concentrations of CAHs, electrical conductivity (EC), DO, ORP, and pH detected at different depths in the MLSWs. The injection of C-MIX combined with reverse circulation leads to the stimulation of dechlorinating activity and the formation of ethylene. The degradation rate of total CAHs at horizons impacted by significant contaminant concentrations (MLSW2A) is 548 μg l^−1^ d^−1^. The subsequent rebound effect, highlighted by the increase in CAHs concentrations, results from the mass flow of mobilized contaminants from the recirculation of water around the GCW. The rebound of chloroethanes may arise from back-diffusion phenomena and dissolution of recalcitrant DNAPL (West and Kueper, 2012). This may be enhanced by the increased concentration gradients generated by the removal of dissolved chlorinated solvents following treatment (Hunkeler et al., 2003). The circulation causes the transport of more distant pollutants into the bioreactive zone near the well, where they are continuously degraded. The decrease of contaminant concentrations over time testifies the stimulation of dechlorinating activity throughout the treatment area and the generation of a subsurface dechlorinating biological reactor. Similar to Schaefer et al. (2018), the findings point to the persistence of long-term attenuation processes, impact on DNAPL rebound mass, and complete enhanced biotic reductive dechlorination.

**Figure 7:**
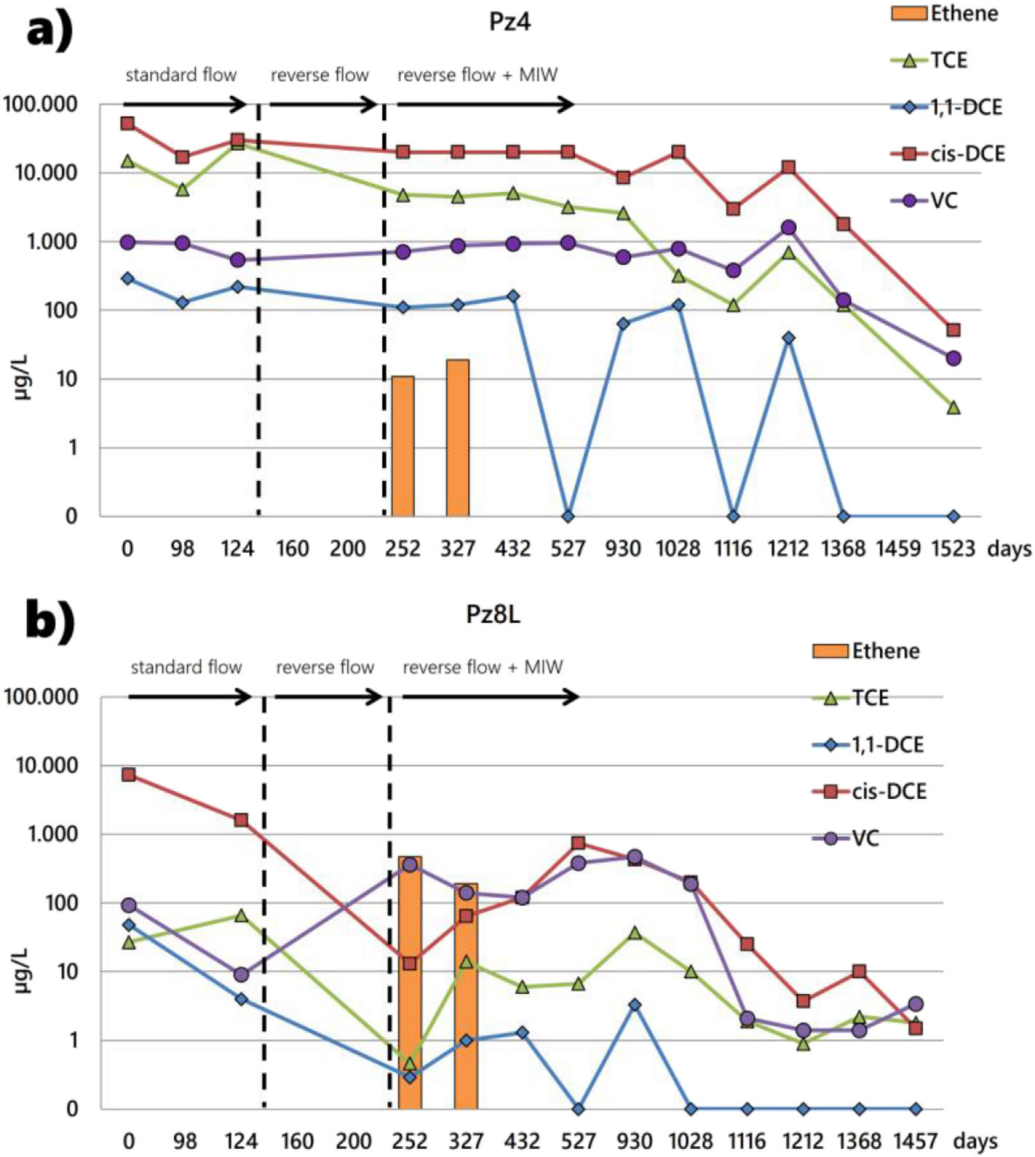
CAH concentrations (log scale) in Pz4 (a) and Pz8L (b) during different remediation phases.

Analyses from traditional and completely screened monitoring piezometers provide some additional insights into the decontamination dynamics in a contaminated heterogeneous and multi-layer aquifer. Pz4 intercepts the shallow aquifer and is located 18 m from the GCW and 6 m from MIW4. It does not show any mobilization triggered by the recirculation but indicates ethylene production related to nutrient injection and gradual decline in CAHs concentrations (Figure 7a and Table S10 of Supplementary Material). The PZ8L intercepts the deep aquifer and is located in the treatment area. Trends in organochlorine concentrations exhibit the reduction in pollutant concentration for standard and reverse flow induced in the shallow aquifer. In analogy to MLSWs, ethylene formation is associated with the injection of C-MIX into the shallow saturated level and is followed first by a rebound effect and then by decreasing CAH concentrations. This evidence suggests the influence of recirculation and the impact of the remediation system on the deeper aquifer, groundwater mixing, and hydraulic communication of aquifer bodies (Figure 7b and Table S11 of Supplementary Material).

The whole set of findings demonstrates that the GCW acts as a 3D distributor of nutrients in the aquifer and reveals both the stimulation of the dechlorinating biological activity and the reduction of chlorinated solvent concentrations in groundwater during remediation time. The GCW-MIWs coupled strategy addresses the limitations evidenced by other carbon substrate addition systems, associated with geological heterogeneity, limited zones of influence near injection points, and non-uniformly distributed biostimulation (Dyer et al., 2006; Manoli et al., 2012). Besides, periodic monitoring of the nutrient redistribution system and the piezometric network indicates that microbial growth does not cause appreciable clogging of aquifer sediments (Adamson and Newell, 2009). Groundwater recirculation may minimize pore clogging and limited migration/distribution efficiency.

### 3.4 The isotopic signature unmasking biodegradation dynamics

**Figure 8:**
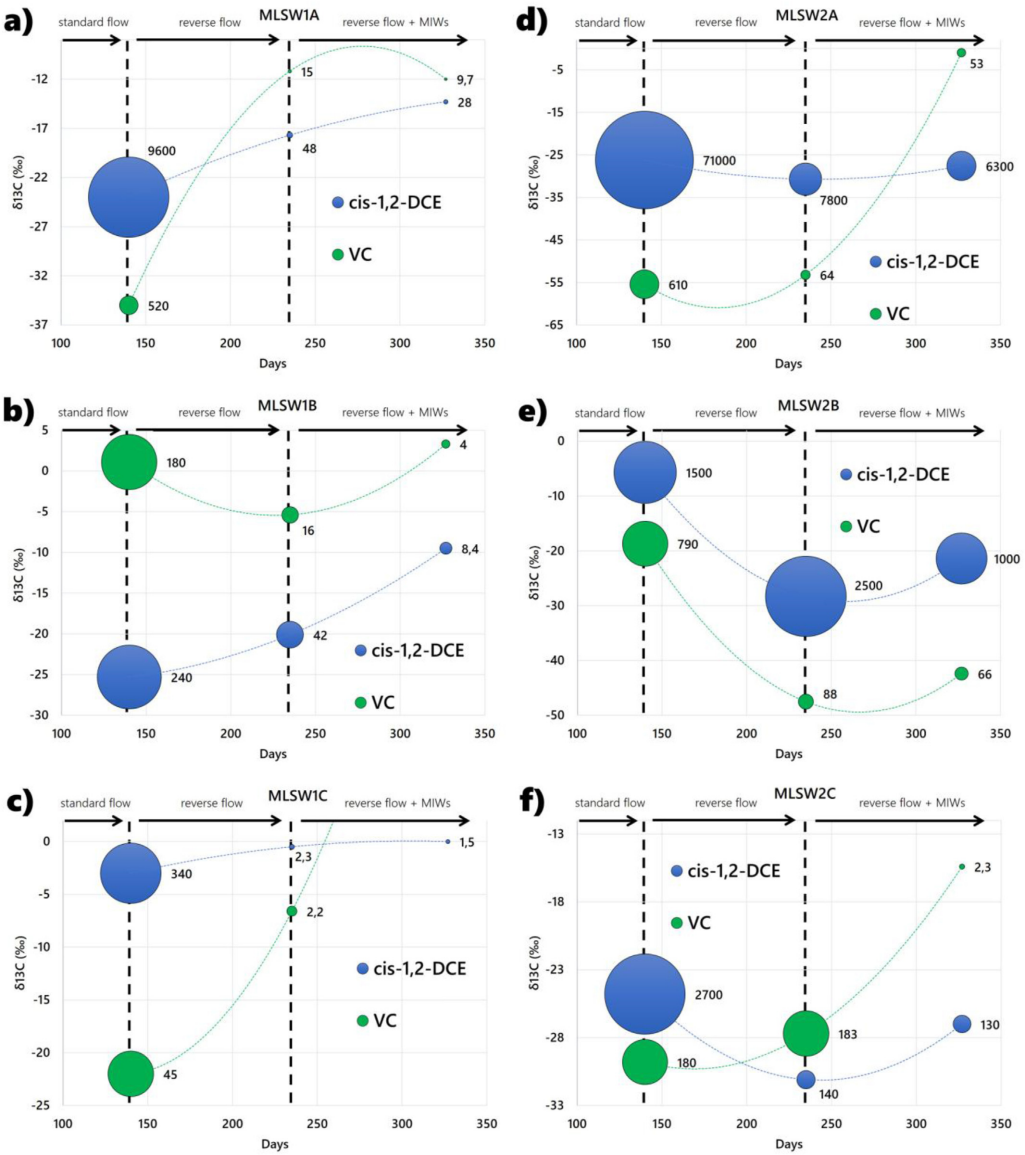
The bubble charts represent the concentrations and stable carbon isotope ratios of cis-1,2-DCE and VC in groundwater for different GCW flow variations during remediation time and different vertical depths of MLSW 1 (a, b, c) and 2 (d, e, f). The size of the bubbles is proportional to the detected concentrations, the labels next to the bubbles indicate the concentration in μg/l.

The sampling point MLSW 1 located in the vicinity of the GCW shows after about 90 days a ^13^C isotopic enrichment of VC and cis-1,2-DCE. This trend is amplified after the addition of C-Mix (nutrients) via the peripheral injection wells from day 240 on (Figure 8a, b, and c). This process becomes also visible in MLWS 2 with some delay because non degraded lighter isotopes are drawn in the first months into the GCW (Figure 8d, e, and f). The overall contaminant reduction in groundwater can be definitively linked to microbiological degradation processes.

Carbon isotopic fractionation has been observed during dechlorination of chlorinated ethenes by indigenous bacteria at various field sites (Bloom et al., 2000; Slater et al., 2001; Hunkeler et al., 2005; Nijenhuis et al., 2013; Filippini et al. 2016). In harmony with the findings of previous studies, the general increasing trend of δ^13^C is associated with enhanced biodegradation. This is favored by standard and reverse recirculation and enhanced by nutrient injection. The average δ^13^C values of cis-1,2-DCE and VC correspond to **−**18.2% and **−**26.6% at the end of the standard recirculation stage. Reverse recirculation reveals average δ^13^C values of cis-1,2-DCE and VC of **−**21.4% and **−**25.3%, respectively. Consistent with the literature, the local drops in δ13C values of cis-1,2-DCE and VC that are induced by recirculation (observed, for example, in Figure 8e) indicate the mobilization and dissolution of chloroethenes from the source area (Song et al., 2002). Nutrient injection results in increased δ^13^C values, averaging **−**16.6% for cis-1,2-DCE and **−**5.4‰ for VC. Post-injection isotope fractionation reveals that the δ^13^C values of cis-1,2-DCE and VC all increased, indicating greater degrees of reductive dichlorination. In addition, the measured chlorinated solvents exhibit an isotopic fingerprint between δ^13^C **−**23 and **−**37%, which may be linked to plumes formed after spills of higher chlorinated commercial primary products such as TCE (Hunkeler et al., 2005; Nijenhuis et al., 2013; Filippini et al. 2016). The monitoring data exhibit a reduction of CAHs concentrations in a relatively short period, considering that time frames for CAH remediation take a long time (Stroo and Ward, 2010; Schaefer et al., 2018). Coupling GCW with C-MIX accelerated the depletion of secondary sources of contamination, mobilizing contaminants, enhancing the plume's biodegradative processes, and inducing long-term persistent dehalorespiring microbial activity. The GCW process's ability to redistribute nutrients in the subsurface reflects the adjustment of well design and screen placement based on site-specific geological and physicochemical site conditions. In this sense, the development of a hydrogeochemical conceptual site model composes a multi-source puzzle governing the installation of an innovative and environmentally sustainable remediation strategy, unmasking the in situ remediation mechanics (physicochemical and microbiological), and helping to better assess remediation actions.

## 4 Conclusions

The hydrogeochemical conceptual model aligns and optimizes the remediation strategy that is tailored to the physicochemical and microbiological conditions and delineates the mechanisms and dynamics of decontamination in the hydrogeological and biochemical context. Modifications of flow direction in the groundwater circulation have a strong influence on groundwater flow pattern and microbial degradation processes. Injection of electron donors like C-MIX through the MIWs in combination with GCW-induced recirculation generates a hydro-bio-geo-chemical reactor and offers a novel and promising strategy to remove CAHs and other contaminants. The multi-source model expanded through CSIA reveals and monitors the in situ transformations of chlorinated solvents. The average δ^13^C values of cis-1,2-DCE at the various stages of the remediation strategy application range from **−**18.2% to 21.4% and then 16.6%. The isotopic enrichment is also evidenced by a gradual increase in the δ^13^C of the VC from **−**26.6% to **−**25.3% and then **−**5.4%, during the application of the intervention at the field scale. The local drops in δ^13^C values that are induced by recirculation reveal the mobilization and dissolution of chloroethenes from the source area. Post-injection isotope fractionation shows that all the δ^13^C values increased, indicating greater degrees of reductive dichlorination. The GCW-MIWs coupled strategy increases the degradation rate and the extension of bioactive surface in the circulation area through enhanced three-dimensional mixing of groundwater, contaminants, microorganisms, and biostimulants. The degradation rate of total CAHs locally amounts to 548 μg l^−1^ d^−1^. The GCW system improves both the bioavailability of pollutants by mobilizing and solubilizing secondary CAH contamination sources and the 3D spread of additives throughout the treatment area. Evidence from the field experiment suggests permanent stimulation and persistence of dechlorinating microbiological activity and supports a rapid decline in CAHs concentrations in all horizontal and vertical areas of the aquifer. The injection and distribution mechanism can be extended to numerous other reagents by varying the recirculation and injection configuration.

## Declarations

### Author contribution statement

Paolo Ciampi: Conceived and designed the experiments; Analyzed and interpreted the data; Wrote the paper.

Carlo Esposito: Conceived and designed the experiments; Analyzed and interpreted the data.

Ernst Bartsch: Conceived and designed the experiments; Performed the experiments; Analyzed and interpreted the data; Contributed reagents, materials, analysis tools or data.

Eduard J. Alesi: Conceived and designed the experiments; Performed the experiments; Analyzed and interpreted the data; Contributed reagents, materials, analysis tools or data; Wrote the paper.

Gert Rehner: Conceived and designed the experiments; Performed the experiments; Contributed reagents, materials, analysis tools or data.

Marco Petrangeli Papini: Conceived and designed the experiments; Analyzed and interpreted the data.

### Funding statement

This research did not receive any specific grant from funding agencies in the public, commercial, or not-for-profit sectors.

### Data availability statement

Data included in article/supp. material/referenced in article.

### Declaration of interest’s statement

The authors declare no conflict of interest.

### Additional information

Supplementary content related to this article has been published online at [URL].

## Appendix A Supplementary data

Revised Supplementary Material - highlighting revisions made Revised Supplementary Material - highlighting revisions made

## References

[bib1] Adamson D.T., Newell C.J. (2009). Support of source zone bioremediation through endogenous biomass decay and electron donor recycling. Biorem. J.

[bib2] Amanat N., Matturro B., Rossi M.M., Valentino F., Villano M., Petrangeli Papini M. (2021). Assessment of long-term fermentability of PHA-based materials from pure and mixed microbial cultures for potential environmental applications. Water.

[bib3] Anam G.B., Choi J., Ahn Y. (2019). Reductive dechlorination of perchloroethene (PCE) and bacterial community changes in a continuous-flow, two-stage anaerobic column. Int. Biodeterior. Biodegrad..

[bib4] Anderson M.R., Johnson R.L., Pankow J.F. (1992). Dissolution of dense chlorinated solvents into ground water: 1. Dissolution from a well-defined residual source. Ground Water.

[bib5] Aulenta F., Pera A., Rossetti S., Petrangeli Papini M., Majone M. (2007). Relevance of side reactions in anaerobic reductive dechlorination microcosms amended with different electron donors. Water Res..

[bib6] Blázquez-Pallí N., Rosell M., Varias J., Bosch M., Soler A., Vicent T., Marco-Urrea E. (2019). Multi-method assessment of the intrinsic biodegradation potential of an aquifer contaminated with chlorinated ethenes at an industrial area in Barcelona (Spain). Environ. Pollut..

[bib7] Bloom Y., Aravena R., Hunkeler D., Edwards E., Frape S. (2000). Carbon isotope fractionation during microbial dechlorination of trichloroethene, cis-1, 2-dichloroethene, and vinyl chloride: implications for assessment of natural attenuation. Environ. Sci. Technol..

[bib8] Bott-Breuning G., Alesi E.J. (1993). Biologische In-Situ Sanierung durch Grundwasserzirkulation mit dem Unterdruck Verdampfer Brunnen (UVB). L. Schimmelpfeng (Hrsg.) Altlasten, Deponietechnik, Kompostierung 1993. Acad. Verlag.

[bib9] Brusseau M.L., Guo Z. (2014). Assessing contaminant-removal conditions and plume persistence through analysis of data from long-term pump-and-treat operations. J. Contam. Hydrol..

[bib10] Chen F., Li Z.L., Yang J.Q., Liang B., Lin X.Q., Nan J., Wang A.J. (2018). Effects of different carbon substrates on performance, microbiome community structure and function for bioelectrochemical-stimulated dechlorination of tetrachloroethylene. Chem. Eng. J..

[bib11] Ciampi P., Esposito C., Petrangeli Papini M. (2019). Hydrogeochemical model supporting the remediation strategy of a highly contaminated industrial site. Water.

[bib12] Ciampi P., Esposito C., Viotti P., Boaga J., Cassiani G., Petrangeli Papini M. (2019). An integrated approach supporting remediation of an aquifer contaminated with chlorinated solvents by a combination of adsorption and biodegradation. Appl. Sci..

[bib13] Ciampi P., Esposito C., Bartsch E., Alesi E.J., Petrangeli Papini M. (2021). 3D dynamic model empowering the knowledge of the decontamination mechanisms and controlling the complex remediation strategy of a contaminated industrial site. Sci. Total Environ..

[bib14] Ciampi P., Esposito C., Cassiani G., Deidda G.P., Rizzetto P., Petrangeli Papini M. (2021). A field-scale remediation of residual light non-aqueous phase liquid (LNAPL): chemical enhancers for pump and treat. Environ. Sci. Pollut. Res..

[bib15] Ciampi P., Esposito C., Cassiani G., Deidda G.P., Flores-Orozco A., Rizzetto P., Chiappa A., Bernabei M., Gardon A., Petrangeli Papini M. (2022). Contamination presence and dynamics at a polluted site: spatial analysis of integrated data and joint conceptual modeling approach. J. Contam. Hydrol..

[bib16] Ciampi P., Esposito C., Bartsch E., Alesi E.J., Nielsen C., Ledda L., Lorini L., Petrangeli Papini M. (2022). Coupled hydrogeochemical approach and sustainable technologies for the remediation of a chlorinated solvent plume in an urban area. Sustainability.

[bib17] Dyer M., Marnette E., Schuren C., van den Brink K. (2006). Full-scale stimulation of reductive dechlorination using the Liner® technique. Eng. Geol..

[bib18] Dolinová I., Strojsová M., Cernik M., Nemecek J., Machacková J., Sevcu A. (2017). Microbial degradation of chloroethenes: a review. Environ. Sci. Pollut. Res..

[bib19] Filbà M., Salvany J.S., Jubany J., Carrasco L. (2016). Tunnel boring machine collision with an ancient boulder beach during the excavation of the Barcelona city subway L10 line: a case of adverse geology and resulting engineering solutions. Eng. Geol..

[bib20] Filippini M., Amorosi A., Campo B., Herrero-Martìn S., Nijenhuis I., Parker B.L., Gargini A. (2016). Origin of VC-only plumes from naturally enhanced dechlorination in a peat-rich hydrogeologic setting. J. Contam. Hydrol..

[bib21] Flores Orozco F., Ciampi P., Katona T., Censini M., Petrangeli Papini M., Deidda G.P., Cassiani G. (2021). Delineation of hydrocarbon contaminants with multi-frequency complex conductivity imaging. Sci. Total Environ..

[bib22] Guan X., Du X., Liu M., Qin H., Qiao J., Sun Y. (2020). Enhanced trichloroethylene dechlorination by carbon-modified zero-valent iron: revisiting the role of carbon additives. J. Hazard..

[bib23] He H., Li Y., Shen R., Shim H., Zeng Y., Zhao S., Lu Q., Mai B., Wang S. (2021). Environmental occurrence and remediation of emerging organohalides: a review. Environ. Pollut..

[bib24] Herrero J., Puigserver D., Nijenhuis I., Kuntze K., Parker B.L., Carmona J.M. (2021). The role of ecotones in the dehalogenation of chloroethenes in alluvial fan aquifers. Environ. Sci. Pollut. Res..

[bib25] Herrling B., Stamm J., Buermann W., Hinchee R.E., Olfenbuttel R.F. (1991). In Situ Bioreclamation.

[bib26] Herrling B., Stamm J., Alesi E.J., Brinnel P., Hirschberger F., Sick M.R. (1991). Proceedings, Third Forum on Innovative Hazardous Waste Treatment Technologies: Domestic and International.

[bib27] Herrling B., Stamm J., Alesi E.J. (1993). Proc. "Environmental Remediation Conference", October 24-28, 1993.

[bib28] Herrling B., Stamm J., Alesi E.J. (1993).

[bib29] Hunkeler D., Aravena R., Parker B.L., Cherry J.A., Diao X. (2003). Monitoring oxidation of chlorinated ethenes by permanganate in groundwater using stable isotopes: dual carbon-chlorine and field studies. Environ. Sci. Technol..

[bib30] Hunkeler D., Aravena R., Berry-Spark K., Cox E. (2005). Assessment of degradation pathways in an aquifer with mixed chlorinated hydrocarbon contamination using stable isotope analysis. Environ. Sci. Technol..

[bib31] Kueper B.H., Stroo H.F., Vogel C.M., Ward C.H. (2014).

[bib32] Laor Y., Ronen D., Graber E.R. (2003). Using a passive multi-layer sampler for measuring detailed profiles of gas-phase VOCs in the unsaturated zone. Environ. Sci. Technol..

[bib33] Manoli G., Chambon J.C., Bjerg P.L., Scheutz C., Binning P.J., Broholm M.M. (2012). A remediation performance model for enhanced metabolic reductive dechlorination of chloroethenes in fractured clay till. J. Contam. Hydrol..

[bib34] Matturro B., Ubaldi C., Rossetti S. (2016). Microbiome dynamics of a polychlorobiphenyl (PCB) historically contaminated marine sediment under conditions promoting reductive dechlorination. Front. Microbiol..

[bib35] Mateas D.J., Tick G.R., Carroll K.C. (2017). In situ stabilization of NAPL contaminant source-zones as a remediation technique to reduce mass discharge and flux to groundwater. J. Contam. Hydrol..

[bib36] Merlino G., Balloi A., Marzorati M., Mapelli F., Rizzi A., Lavazza D., de Ferra F., Carpani G., Daffonchio D. (2015). Diverse reductive dehalogenases are associated with clostridiales-enriched microcosms dechlorinating 1,2-dichloroethane. BioMed Res. Int..

[bib37] Němeček J., Dolinová I., Macháčková J., Špánek R., Ševců A., Lederer T., Černík M. (2017). Stratification of chlorinated ethenes natural attenuation in an alluvial aquifer assessed by hydrochemical and biomolecular tools. Chemosphere.

[bib38] Nijenhuis I., Schmidt M., Pellegatti E., Paramatti E., Richnow H.H., Gargini A. (2013). A stable isotope approach for source apportionment of chlorinated ethene plumes at a complex multi-contamination events urban site. J. Contam. Hydrol..

[bib39] Niño de Guzmán G.T., Hapeman C.J., Millner P.D., Torrents A., Jackson D., Kjellerup B.V. (2018). Presence of organohalide-respiring bacteria in and around a permeable reactive barrier at a trichloroethylene-contaminated Superfund site. Environ. Pollut..

[bib40] Petrangeli Papini M., Majone M., Arjmand F., Silvestri D., Sagliaschi M., Sucato S., Alesi E. (2016). First pilot test on integration of gcw (groundwater circulation well) with ena (enhanced natural attenuation) for chlorinated solvents source remediation. Chem. Eng. Trans..

[bib41] Pierro L., Matturro B., Rossetti S., Sagliaschi M., Sucato S., Alesi E., Bartsch E., Arjmand F., Petrangeli Papini M. (2017). Polyhydroxyalkanoate as a slow-release carbon source for in situ bioremediation of contaminated aquifers: from laboratory investigation to pilot-scale testing in the field. N. Biotech..

[bib42] Puigserver D., Herrero J., Torres M., Cortés A., Nijenhuis I., Kuntze K., Parker B.L., Carmona J.M. (2016). Reductive dechlorination in recalcitrant sources of chloroethenes in the transition zone between aquifers and aquitards. Environ. Sci. Pollut. Res..

[bib43] Puigserver D., Herrero J., Parker B.L., Carmona J.M. (2020). Natural attenuation of pools and plumes of carbon tetrachloride and chloroform in the transition zone to bottom aquitards and the microorganisms involved in their degradation. Sci. Total Environ..

[bib44] Puigserver D., Herrero J., Nogueras X., Cortés A., Parker B.L., Playà E., Carmona J.M. (2022). Biotic and abiotic reductive dechlorination of chloroethenes in aquitards. Sci. Total Environ..

[bib45] Rajajayavel S.R.C., Ghoshal S. (2015). Enhanced reductive dechlorination of trichloroethylene by sulfidated nanoscale zerovalent iron. Water Res..

[bib46] Renard P., Allard D. (2013). Connectivity metrics for subsurface flow and transport. Adv. Water Resour..

[bib47] Rossi M.M., Dell’Armi E., Lorini L., Amanat N., Zeppilli M., Villano M., Petrangeli Papini M. (2021). Combined strategies to prompt the biological reduction of chlorinated aliphatic hydrocarbons: new sustainable options for bioremediation application. Bioengineering.

[bib48] Schaefer C.E., Lavorgna G.M., Haluska A.A., Annable M.D. (2018). Long-term impacts on groundwater and reductive dechlorination following bioremediation in a highly characterized trichloroethene DNAPL source area. Groundwater Monit. R..

[bib49] Slater G.F., Sherwood Lollar B., Sleep B.E., Edwards E.A. (2001). Variability in carbon isotopic fractionation during biodegradation of chlorinated ethenes: implications for field applications. Environ. Sci. Technol..

[bib50] Song D.L., Conrad M.E., Sorenson K.S., Alvarez-Cohen L. (2002). Stable carbon isotope fractionation during enhanced in situ bioremediation of trichloroethene. Environ. Sci. Technol..

[bib51] Stroo H.F., Ward C.H. (2010).

[bib52] Stamm J. (1997). Numerische Berechnung dreidimensionaler Strömungsvorgänge um Grundwasser Zirkulations Brunnen zur In situ Grundwassersanierung. Fortschritt Berichte VDI, Reihe.

[bib53] Tatti F., Petrangeli Papini M., Torretta V., Mancini G., Boni M.R., Viotti P. (2019). Experimental and numerical evaluation of Groundwater Circulation Wells as a remediation technology for persistent, low permeability contaminant source zones. J. Contam. Hydrol..

[bib54] Utom A.U., Werban U., Leven C., Müller C., Dietrich P. (2019). Adaptive observation-based subsurface conceptual site modeling framework combining interdisciplinary methodologies: a case study on advancing the understanding of a groundwater nitrate plume occurrence. Environ. Sci. Pollut. Res..

[bib55] Wang S.Y., Kuo Y.C., Huang Y.Z., Huang C.W., Kao C.M. (2015). Bioremediation of 1,2-dichloroethane contaminated groundwater: microcosm and microbial diversity studies. Environ. Pollut..

[bib56] Wang X., Xin J., Yuan M., Zhao F. (2020). Electron competition and electron selectivity in abiotic, biotic, and coupled systems for dechlorinating chlorinated aliphatic hydrocarbons in groundwater: a review. Water Res..

[bib57] West M.R., Kueper B.H. (2012). Numerical simulation of DNAPL source zone remediation with in situ chemical oxidation (ISCO). Adv. Water Resour..

[bib58] Wycisk P., Stollberg R., Neumann C., Gossel W., Weiss H., Weber R. (2013). Integrated methodology for assessing the HCH groundwater pollution at the multi-source contaminated mega-site Bitterfeld/Wolfen. Environ. Sci. Pollut. Res..

[bib59] Xia Q., Zhang Q., Xu M., Tang Y., Teng H. (2019). Visualizing hydraulic zones of a vertical circulation well in presence of ambient flow. Desalination Water Treat..

[bib60] Xiao Z., Jiang W., Chen D., Xu Y. (2020). Bioremediation of typical chlorinated hydrocarbons by microbial reductive dechlorination and its key players: a review. Ecotoxicol. Environ. Saf..

[bib61] Yang L., Wang X., Mendoza-Sanchez I., Abriola L.M. (2018). Modeling the influence of coupled mass transfer processes on mass flux downgradient of heterogeneous DNAPL source zones. J. Contam. Hydrol..

[bib62] Ye Y., Zhang Y., Lu C., Xie Y., Luo J. (2021). Effective chemical delivery through multi-screen wells to enhance mixing and reaction of solute plumes in porous media. Water Resour. Res..

[bib63] You X., Liu S., Dai C., Guo Y., Zhong G., Duan Y. (2020). Contaminant occurrence and migration between high- and low-permeability zones in groundwater systems: a review. Sci. Total Environ..

