# Supplementary material for: Remediation of chlorinated aliphatic hydrocarbons (CAHs) contaminated site coupling groundwater recirculation well (IEG-GCW®) with a peripheral injection of soluble nutrient supplement (IEG-C-MIX) via multilevel-injection wells (IEG-MIW)
Source: Heliyon. 2022 Nov 3;8(11):e11402. doi: 10.1016/j.heliyon.2022.e11402 (PMC9647460; doi:10.1016/j.heliyon.2022.e11402)
Supplement: Revised Supplementary Material - highlighting revisions made [file mmc1.doc]

Supplementary Material


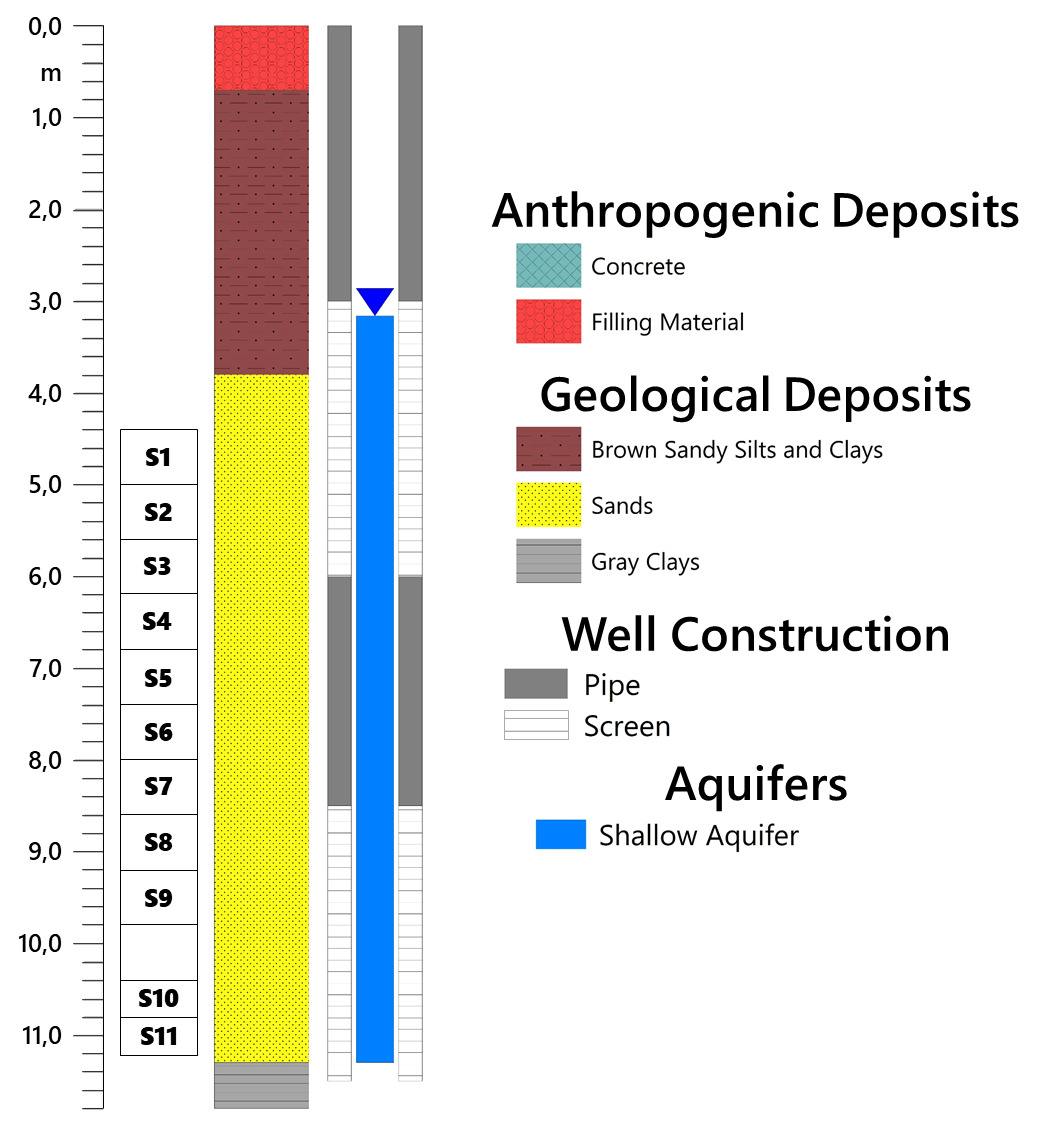


Fig. S1. Core samples collected at different depths for grain size analysis along the vertical of the IEG-GCW borehole. Schematic representation of the stratigraphy and construction scheme of the recirculation well.


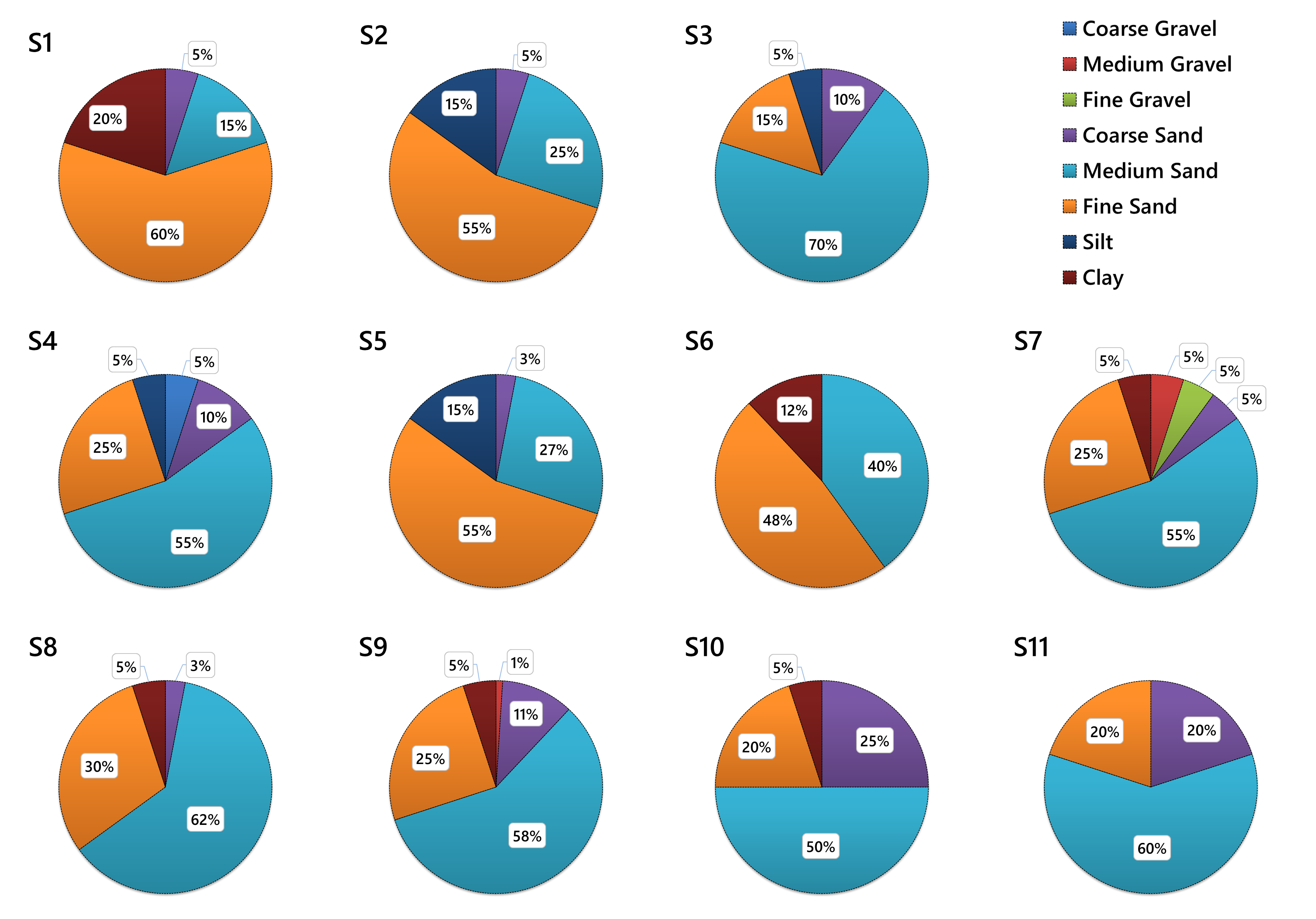


Fig. S2. Pie charts summarizing the percent ratio of granulometric fractions by referencing particle size limits of 20 mm (coarse-medium gravel), 5 mm (medium-fine gravel), 2 mm (fine gravel-coarse sand), 0.5 mm (coarse-medium sand), 0.25 mm (medium-fine sand), 50 μm (fine sand-silt), and 2 μm (clay-silt). Labels S1-S11 refer to the eleven core samples collected for particle size analysis along the vertical of the IEG-GCW borehole.

| Sample ID | Sampling depth interval (m) | k (m/s) |
| --- | --- | --- |
| S1 | 4,4 - 5,0 | 1,44 x 10^-5^ |
| S2 | 5,0 - 5,6 | 2,23 x 10^-5^ |
| S3 | 5,6 - 6,2 | 1,66 x 10^-4^ |
| S4 | 6,2 - 6,8 | 1,67 x 10^-4^ |
| S5 | 6,8 - 7,2 | 4,67 x 10^-5^ |
| S6 | 7,2 - 7,8 | 8,26 x 10^-5^ |
| S7 | 7,8 - 8,6 | 1,37 x 10^-4^ |
| S8 | 8,6 - 9,2 | 1,72 x 10^-4^ |
| S9 | 9,2 - 9,8 | 1,67 x 10^-4^ |
| S10 | 10,4 - 10,8 | 1,86 x 10^-4^ |
| S11 | 10,8 - 11,2 | 1,90 x 10^-4^ |

Tab. S1. Estimation of permeability coefficient (k) from particle size analysis on core samples collected at different depths during characterization activities.

| Piezometer | 1,1-DCE (µg/l) | cis-DCE (µg/l) | TCE (µg/l) | VC (µg/l) | Total CAHs (µg/l) |
| --- | --- | --- | --- | --- | --- |
| PZ1 | 320 | 13000 | 170000 | 0 | 183320 |
| PZ2 | 71 | 13000 | 930 | 120 | 14121 |
| PZ3 | 17 | 13000 | 450 | 33 | 13500 |
| PZ4 | 7,1 | 790 | 820 | 41 | 1658,1 |
| PZ5 | 6,2 | 760 | 11 | 4 | 781,2 |
| PZ6 | 11 | 2400 | 540 | 41 | 2992 |
| PZ7 | 180 | 8600 | 61000 | 130 | 69910 |

Tab. S2. Concentrations of CAHs detected in the pre-remediation monitoring campaign in the piezometric network.

Tab. S3. Concentrations of CAHs detected in the pre-remediation monitoring campaign in several soil samples collected at different depths.


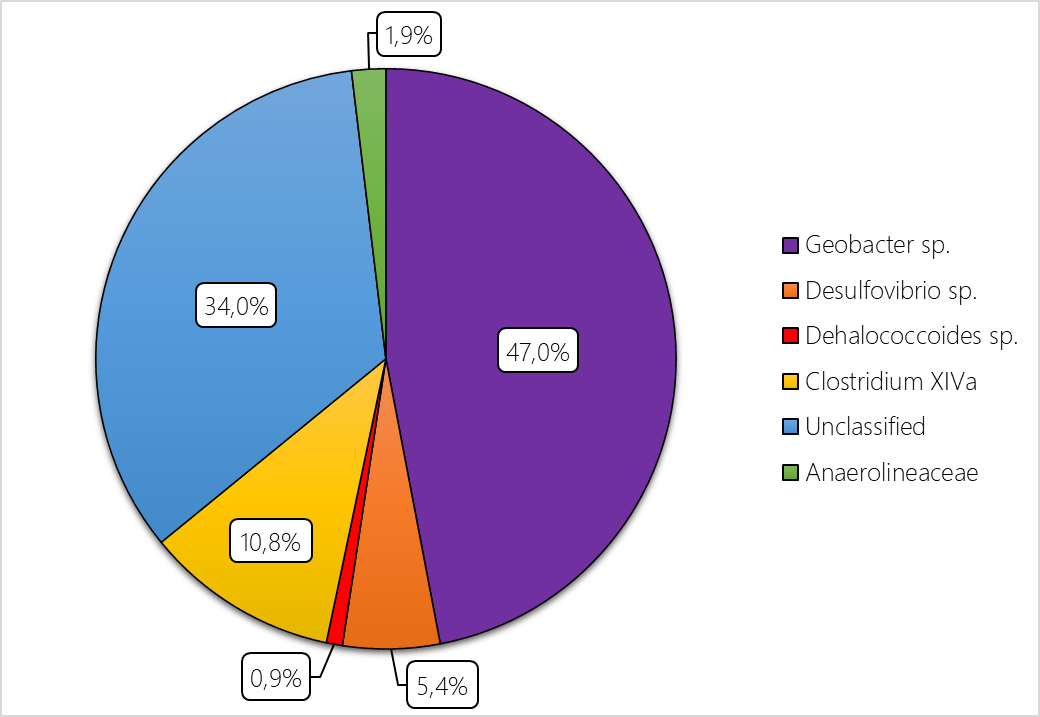


Fig. S3. Microbial characterization and taxonomic assignment resulting from NGS experiments of bacterial 16S rDNA in microcosm test.


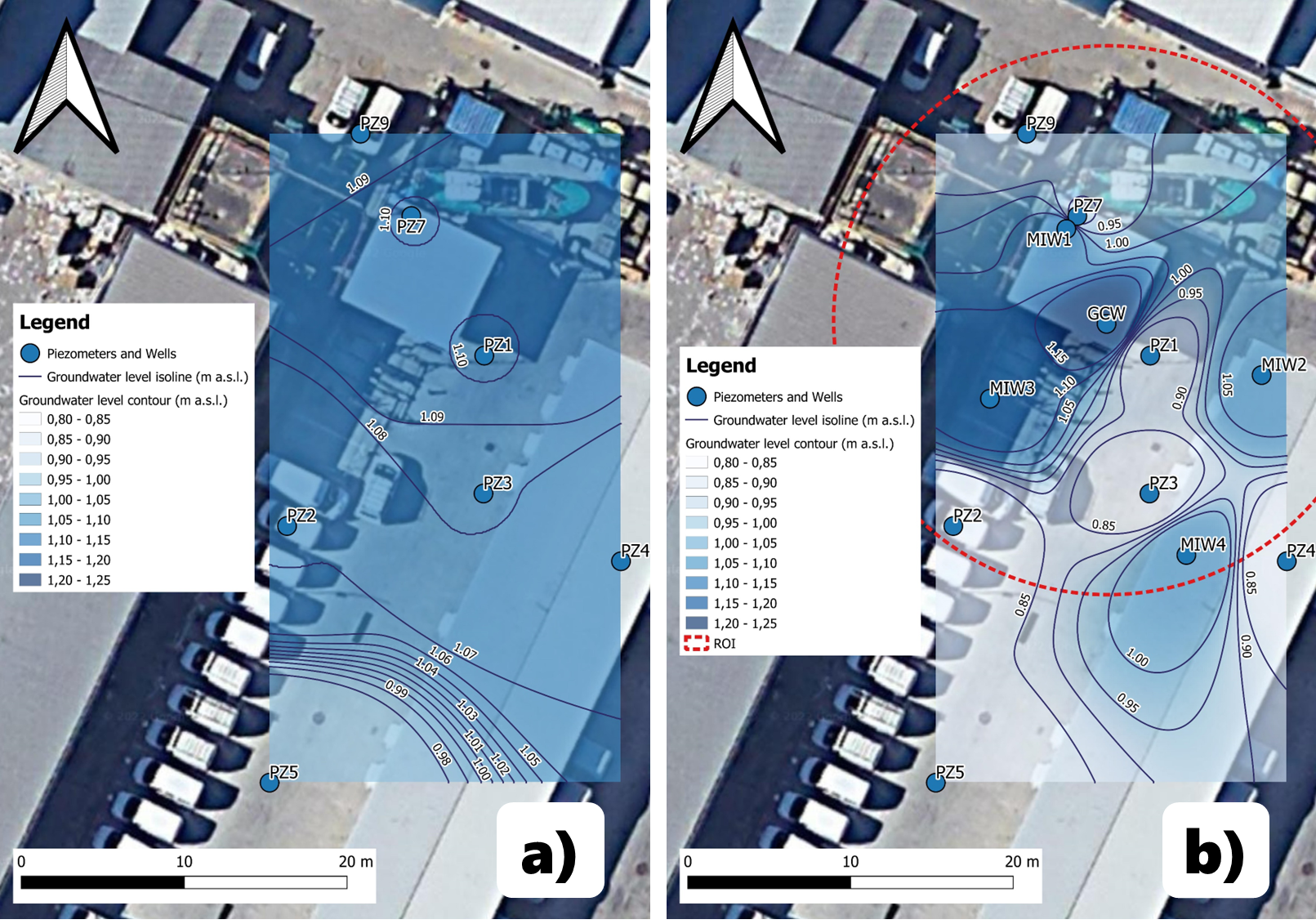


Fig. S4. Comparison of groundwater levels in the pre-remediation phase (a) and following GCW activation in standard-flow mode (b).

Tab. S4. Concentrations of CAHs, electrical conductivity (EC), dissolved oxygen (DO), oxidation-reduction potential (ORP), and pH detected during remediation time at MLSW1A.

Tab. S5. Concentrations of CAHs, electrical conductivity (EC), dissolved oxygen (DO), oxidation-reduction potential (ORP), and pH detected during remediation time at MLSW1B.

Tab. S6. Concentrations of CAHs, electrical conductivity (EC), dissolved oxygen (DO), oxidation-reduction potential (ORP), and pH detected during remediation time at MLSW1C.

Tab. S7. Concentrations of CAHs, electrical conductivity (EC), dissolved oxygen (DO), oxidation-reduction potential (ORP), and pH detected during remediation time at MLSW2A.

Tab. S8. Concentrations of CAHs, electrical conductivity (EC), dissolved oxygen (DO), oxidation-reduction potential (ORP), and pH detected during remediation time at MLSW2B.

Tab. S9. Concentrations of CAHs, electrical conductivity (EC), dissolved oxygen (DO), oxidation-reduction potential (ORP), and pH detected during remediation time at MLSW2C.

Tab. S10. Concentrations of CAHs, electrical conductivity (EC), dissolved oxygen (DO), oxidation-reduction potential (ORP), and pH detected during remediation time at PZ4.

Tab. S11. Concentrations of CAHs, electrical conductivity (EC), dissolved oxygen (DO), oxidation-reduction potential (ORP), and pH detected during remediation time at PZ8L.
